# Extracellular Vesicles in Tauopathies: Mechanisms and Applications

**DOI:** 10.3390/ijms27041998

**Published:** 2026-02-19

**Authors:** Varun Nannuri, Ebaa Ababneh, Serena El Rayes, Jonhoi Smith, Kiminobu Sugaya, Jihe Zhao

**Affiliations:** Burnett School of Biomedical Sciences, College of Medicine, University of Central Florida, Orlando, FL 32827, USA

**Keywords:** extracellular vesicles, tauopathy, biomarkers, diagnosis, therapy

## Abstract

Extracellular vesicles (EVs) are nanoscale membrane-enclosed vesicles that mediate intercellular communication and participate in both physiological and pathological signaling processes. Recent studies underscore the critical roles of EVs in the propagation of tau pathology and the diagnostic and therapeutic aspects of tauopathies, a class of neurodegenerative diseases marked by the pathological accumulation of the protein tau, contributing to cognitive decline and neuronal loss. This review aims to explore the many roles of EVs in tauopathies, emphasizing their ability to carry and transmit misfolded tau, modulate immune responses through microglial interactions, and serve as carriers of disease-specific biomarkers. We describe current findings on extravesicular tau, miRNAs, and mRNAs as diagnostic indicators in Alzheimer’s disease and related tauopathies and evaluate the therapeutic potential of both endogenous and engineered EVs for delivering therapeutic agents or neuroprotective cargo. Challenges such as isolation standardization, cargo loading efficiency, and biodistribution are also discussed, along with potential strategies to overcome them. Overall, the evidence emphasizes EVs as key players in the pathophysiology, diagnosis, and treatment of tauopathies, offering a strong and compelling platform for precision medicine and clinical applications in the future.

## 1. Introduction

Extracellular vesicles (EVs) have become known as key mediators of intercellular communication and participate in both normal physiological regulation and pathological signaling in NDs, particularly the tauopathies. EVs have been increasingly recognized as major contributors to disease propagation by transferring misfolded or hyperphosphorylated tau species between connected neurons and glial cells. Their molecular cargo reflects cellular stress responses, synaptic injury, and neuroinflammatory activation, underscoring their value as diagnostic indicators and potential therapeutic vehicles. “EVs” is a general term that refers to all membrane-enclosed vesicles secreted by cells, such as exosomes, microvesicles (ectosomes), and other vesicle subtypes, that differ in size, cellular origin, and/or biogenesis. Nonetheless, the literature frequently describes exosomes based on the vesicles’ size (30 to 150 nm in diameter) and/or the enrichment of commonly used markers, rather than experimental demonstration of endosomal origin, which is an essential part of exosomes’ definition. To address this ambiguity, the International Society for Extracellular Vesicles (ISEV) has provided a standardized set of guidelines (Minimal Information for Studies of Extracellular Vesicles guidelines) that help maintain consistent and accurate terminology use [1]. In this review, consistent with ISEV guidelines and the minimal information for studies of extracellular vesicles (MISEV2023) [1], vesicles are referred to as EVs unless their endosomal origin is definitively established.

Tauopathies are a class of neurodegenerative disorders (NDs) characterized by the pathological accumulation of abnormal tau proteins in the brain. Tau, a microtubule-associated protein, plays a vital role in stabilizing the cytoskeleton of neurons, but in tauopathies, tau undergoes hyperphosphorylation, detaches from microtubules, and aggregates into insoluble inclusions, which contribute to neurodegeneration and clinical symptoms such as cognitive decline and motor dysfunction [2]. Tauopathies are typically categorized into primary tauopathies, where tau pathology is the predominant feature, and secondary tauopathies, where it coexists with other pathologies, such as amyloid-β plaques in Alzheimer’s disease (AD) [3]. Some examples of primary tauopathies include Pick’s disease, progressive supranuclear palsy (PSP), corticobasal degeneration (CBD), globular glial tauopathy, and primary age-related tauopathy (PART) [2]. Alzheimer’s disease, though primarily associated with amyloid plaques, also features extensive tau pathology and is thus considered a secondary tauopathy. These diseases mainly differ in terms of the tau isoforms involved (3R, 4R, or both), the brain regions affected, and common clinical presentations [2]. Parkinson’s disease (PD) was not classically considered a tauopathy, but rather a synucleinopathy; however, accumulating evidence suggests the involvement of tau protein in the pathology of PD, which might even precede the α-synuclein (α-syn) pathology, which is the hallmark of PD [4].

In tauopathies, EVs are implicated in the distribution of tau pathology, serving as vehicles for the transfer of misfolded tau between neurons and contributing to the spread of neurodegeneration [5,6]. Moreover, they have been implicated in the transmission and propagation of other pathological proteins involved in tauopathies, such as Aβ and α-syn [7,8]. Interestingly, the very same mechanism that facilitates the spread of pathology also presents a unique clinical opportunity. Since EVs are released into the circulation, they, along with their cargo, offer potential as accessible biomarkers for early and non-invasive diagnosis of tauopathies, as they could reflect the pathological state of the brain [9,10,11]. For instance, studies have shown that EVs derived from patients with AD and PD contain elevated levels of tau, which correlates with the disease’s progression [12,13].

Beyond their diagnostic role, the importance of EVs in tau pathology and progression makes them potential therapeutic targets [14]. Modulating EV biogenesis, release, or uptake may interrupt tau dissemination, while engineered exosomes can deliver therapeutic cargo directly to target cells, enhancing the efficacy of treatments aimed at mitigating tau pathology [15,16]. This targeted delivery system could potentially improve treatment outcomes by enhancing the bioavailability and specificity of treatments aimed at tau aggregation and neurodegeneration while minimizing off-target effects [17]. As a result, EV-mediated delivery systems are being explored as platforms for precision medicine in tauopathies. The use of EVs as therapeutic tools is thus being explored, and some strategies include engineering EVs to express targeting ligands and/or changing exosome biogenesis to boost their neuroprotective cargo.

Altogether, EVs emerge as central players in the landscape of tauopathies, acting as diagnostic markers, treatment targets, and therapeutic vectors. Their role in pathology, diagnostics, and versatile therapeutic platforms underscores the multifaceted nature of EVs in NDs.

## 2. The Role of EVs in the Pathophysiology of Tauopathies

Tau is a microtubule-associated protein that physiologically stabilizes microtubules in neuronal axons. In the human brain, Tau exists in 6 isoforms due to alternative splicing of the *MAPT* gene. These isoforms differ in terms of N-terminal inserts (0N, 1N, 2N) and the presence of either three (3R) or four (4R) microtubule binding repeats. Interestingly, the level of expression and/or accumulation of these isoforms is linked to pathology of various tauopathies. For example, 4R tauopathies like PSP predominantly have 4R tau aggregates in neurons and glia, while 3R tauopathies such as Pick’s disease predominantly exhibit Pick bodies formed by 3R tau. Mixed 3R+4R tauopathies, including AD, exhibit both isoforms [18]. Tau undergoes post-translational modifications, most commonly phosphorylation. Tau phosphorylation can dictate its fate, microtubule binding, subcellular localization, and signaling pathways in which tau is involved [19,20]. Hyperphosphorylation of tau leads to its aggregation, loss of function, and disrupted clearance [21]. Additionally, tau can be truncated at different residues by proteases. Tau truncation affects tau phosphorylation and thus results in aggregation, misfolding, and their pathological consequences [22]. These phosphorylated or truncated forms, as they accumulate in the cell and escape cellular clearance mechanisms, are sorted into EVs and internalized by recipient cells, which results in their seeding and propagation [5,6,12]. An important observation that connects EVs to tau pathology is that pathogenic tau filaments were found to be physically tethered to the luminal surface of AD brain-derived EVs, which offers structural evidence that tau associates with EVs clinically in postmortem samples [23]. Additionally, evidence indicates that EVs isolated from different human tauopathies (AD, PSP, CBD) carry disease-specific tau species and result in different seeding patterns in vivo. This suggests that EV cargo heterogeneity may be behind the clinical variability in tau pathology progression [24]. EVs from AD patients’ brains caused tau propagation, particularly in the GABAergic interneurons [25], resulting in neuronal dysfunction [25]. This preferential accumulation in interneurons represents an early pathological event that precedes widespread neurodegeneration, indicating the importance of EVs in disease initiation by affecting interneurons [26].

Interestingly, recent structural analysis has shown that tau in EVs is tethered to the internal surface of the vesicle membrane [23], restricting therapeutic accessibility and clearance by immune cells, in addition to disrupting intracellular degradation after uptake.

Neuroinflammation has been increasingly recognized as a key factor in tauopathy, with early-stage inflammation potentially alleviating tau pathology, but later-stage chronic inflammation worsening it [27,28]. A critical intersection between tau propagation and immune response lies in microglial involvement.

Microglia, the immune cells of the central nervous system, play an important role, acting as the immune cells in the brain that support the development of neuronal cells, immune responses, and tissue repair. However, their role can be a detriment and facilitate the progression of neurodegeneration. For example, microglia have been demonstrated to interact with EVs containing tau, facilitating the uptake and propagation of tau aggregates [14,29]. Research indicates that the depletion of microglia can significantly reduce the spread of tau pathology, suggesting that microglial activity is closely linked to EVs’ tau transmission [14]. This highlights the role of microglia as both potential mediators of disease spread and as targets for therapeutic modulation.

Uncontrolled microglial activation is implicated in the progression of NDs, with some studies suggesting inflammation may precede tau pathology in AD. EVs may play a key role in linking tau propagation and microglial activation, with reduced microglial numbers and inhibited exosome synthesis shown to limit tau spread. Phagocytosed tau seeds can trigger inflammasome activation in microglia, promoting chronic inflammation. Collectively, these lines of evidence suggest that the interplay between tau seeding and microglial activation is crucial for tau propagation, offering new avenues for therapeutic and preventative strategies in NDs [30,31].

**Figure 1:**
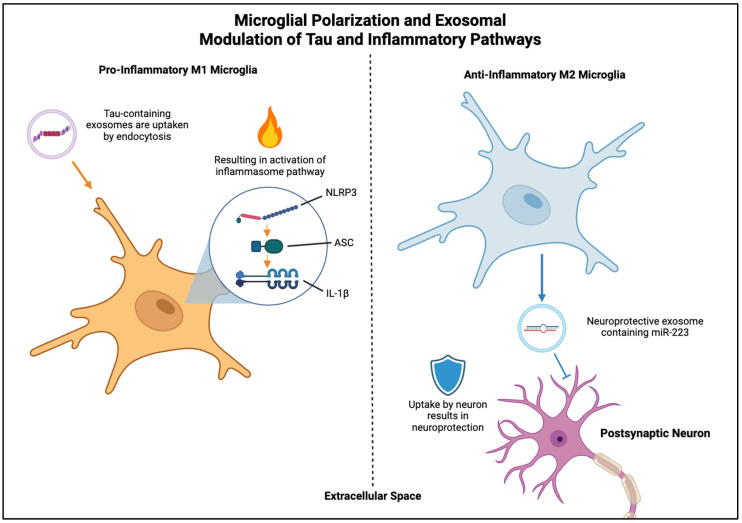
Microglial Polarization and EV Modulation of Tau and Inflammatory Pathways. Pro-inflammatory M1 microglia activate inflammasome signaling via NLRP3–ASC–IL1β pathways, while anti-inflammatory M2 microglia release miR-223-enriched exosomes that protect neurons. Created in BioRender. Zhao, J. (2026) https://BioRender.com/l2y4xt2 (accessed on 11 February 2026).

**Figure 2:**
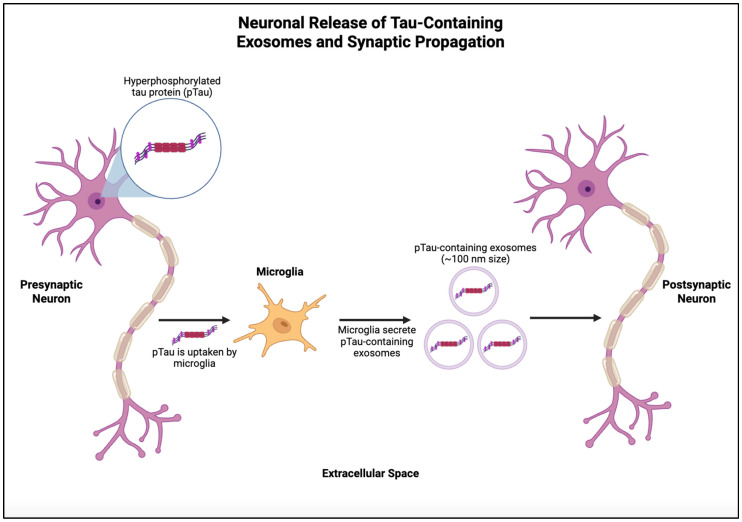
Neuronal release of Tau-containing EVs and synaptic propagation. Microglia uptake pTau released from the presynaptic neurons and subsequently synthesize and secrete pTau-containing EVs, which are taken up by the postsynaptic neurons, facilitating trans neuronal propagation of tau pathology. Created in BioRender. Zhao, J. (2026) https://BioRender.com/l2y4xt2 (accessed on 11 February 2026).

In AD, microglial polarization toward the pro-inflammatory M1 phenotype drives neuroinflammation, as shown in Figure 1 [32]. Conversely, anti-inflammatory M2 microglia can reverse this effect through the interaction of YB-1 with miR-223 and enhanced EVs loading of this miRNA [32], which inhibits neuronal apoptosis [33] and has been identified as a sensitive biomarker for AD [34]. Beyond inflammatory polarization, microglia actively shape tau pathology through mechanisms such as phagocytosis, inflammasome activation, and the secretion of tau-containing EVs. Microglia can internalize both phosphorylated tau (pTau) and Aβ and release them in EVs, promoting transneuronal propagation of pathology, as shown in Figure 2 [14,35,36]. pTau activates interleukin-1β (IL-1β) via myeloid differentiation primary response (MyD88)- and NLRP3-ASC-dependent pathways, linking tau accumulation directly to microglial inflammasome activation and neuroinflammation [37]. Suppressing either pTau or the apoptosis-associated speck-like protein containing a CARD (ASC) has been shown to reduce inflammation and improve cognitive function, underlining the pathological synergy between tau and immune activation [37]. Interestingly, Aβ enhances the seeding activity of tau exosomes [38]. Inflammatory signals induce the cleavage of the Rab-interacting lysosomal protein (RILP), a Rab7 effector involved in lysosomal trafficking, in AD brains and in vitro inflammatory conditions. This resulted in impaired tau degradation and thus the escape and secretion of tau-containing EVs by microglia, highlighting the link between neuroinflammation, tau accumulation, and EV-mediated spread [36].

This pro-pathogenic role is reinforced by evidence that phagocytosed tau seeds can trigger further chronic inflammation via inflammasome signaling. As tau pathology spreads through misfolded conformations along neuronal networks, microglia are increasingly recognized as essential intermediaries in this propagation. Of note, both the reduction in microglial populations and the inhibition of exosome synthesis have been shown to limit tau dissemination, underscoring the critical role of microglia and EVs in this process [39].

Microglia-derived EVs are key mediators of tau spread, which shapes disease trajectory. For example, loss of triggering receptor on myeloid cells 2 (TREM2) exacerbates tau propagation, and this process is EV dependent [39,40]. TREM2 also governs microglial EVs binding to Aβ [41], while purinergic receptor P2X purinoceptor 7 (P2RX7) facilitates tau spread by promoting EV release [42]. These microglial EVs, typically 40–100 nm in size, carry miRNAs, mRNAs, and proteins, and can influence neuronal health positively or negatively depending on their contents and context [32,43], but genetic risk factors further modulate this system. The bridging integrator 1 (*BIN1*) gene, strongly associated with AD risk, has been shown to influence tau pathology, potentially via EV-mediated clearance [44,45], although regional neuroprotective effects vary [46]. TREM2, which was previously discussed, also plays a critical regulatory role, enhancing predictive models of tau propagation when incorporated into computational simulations [47]. Astrocytic and neuron-derived EVs also contribute to the inflammatory and proteopathic landscape. For example, astrocyte-derived exosomes (ADEVs) containing Aβ are enriched in AD brains [48], and neuron-derived exosomes regulate immune responses and contribute to neuroinflammation [49]. Increased secretion of these vesicles under AD conditions reinforces their potential as biomarkers of disease progression and as vehicles for pathological cargo. Autophagy pathways have been implicated in loading these EVs with both Aβ and tau, enhancing their contribution to synaptic dysfunction [50].

Microglia-derived EVs also show promise for therapeutic purposes. For instance, exosomes enriched with miR-223 from anti-inflammatory M2 microglia showed neuroprotective effects [32], while near-infrared (NIR) light therapy promoted the release of microglial EVs containing miR-7670-3p, alleviating endoplasmic reticulum (ER) stress and inflammation [51]. Additionally, EVs derived from microglia have been used to enhance central nervous system (CNS) drug delivery in AD mouse models, resulting in reduced plaque burden and enhanced neuronal recovery [52].

Computational and experimental models continue to refine our understanding of these dynamics. The Nexopathy in silico (Nexis) model, a computational simulation of the progression of tau pathology based on the brain connectome and the neural networks (Nexus), has demonstrated how the inclusion of microglial regulatory genes like TREM2 improves predictions of tau spread, especially to vulnerable brain regions like the hippocampus and striatum [47]. Meanwhile, experiments in tauopathy models confirm that microglia promote tau propagation via EV secretion, with inhibition of EV release curbing disease progression in vitro and in vivo [14]. In parallel, blocking the P2X7 receptor (P2X7R) suppressed microglial tau-laden EV secretion, lowered tau accumulation in vulnerable brain regions, and rescued memory deficits in P301S tauopathy mice [42].

Despite these advances, there remains a gap in understanding the roles of specific microglial subtypes—such as disease-associated microglia (DAM)—in tauopathies. This limits the development of targeted interventions aimed at modulating microglial functions to prevent or treat NDs. Furthermore, a broader gap persists in characterizing the full spectrum of EV–microglial interactions, which likely play pivotal roles in tau propagation and immune responses. Future research must address these unresolved areas to unlock the therapeutic and diagnostic potential of this system.

Neurons release tau through EVs, with exosomal Tau being less phosphorylated than cytosolic Tau. And neuronal activity can enhance this release. The ability of EVs to directly transfer tau between neurons largely depends on the synaptic connectivity [53]. Tau-containing EVs derived from cells or the cerebrospinal fluid (CSF) of AD patients are internalized by neurons and microglia but not astrocytes. Aggregated Tau in neurons can be exported within exosomes, which subsequently triggers inclusion formation in recipient cells expressing mutant Tau. EV-mediated Tau secretion, which can be modulated by disease-related Tau modifications, is suggested to contribute to Tau-induced neurodegeneration. One such study found that Tau in EVs from AD patient CSF is phosphorylated at threonine 181 (Thr-181), a biomarker for AD, and identified proteins co-purified with Tau that are linked to Tau misprocessing. These findings indicate that EV-mediated Tau secretion may play a significant role in Tau processing abnormalities and elevated Tau levels in early AD [12,53].

In AD and related disorders, tau aggregates disrupt neuronal function by forming lesions that impair cell processes. These aggregates spread to neighboring cells, where they corrupt the folding of soluble tau in a prion-like manner, further impairing cellular functions. EVs facilitate the transmission of these tau aggregates. Using a microfluidics culture system with hippocampal neurons, one study utilized two models that were established to track exosome spreading. In the first model, EVs were labeled with fluorescent tags, showing that interconnected neurons exchange EVs only when their axons are in proximity. In the second model, EVs from tau transgenic mice were added to one neuron, revealing that a significant portion was internalized and transferred to a second neuron. This transfer occurred via secretory endosomes, suggesting that EVs hijack the endosomal machinery, enhancing the pathogenic potential of the EVs cargo. Not all internalized EVs were degraded; some persisted, extending the distance over which tau could spread [6]. Synaptic dysfunction, linked to cognitive decline in AD, is driven by tau, but it is unclear whether synapse loss precedes tau accumulation or follows it. Findings from a study show that in a rTgTauEC mouse model, synapse density in the dentate gyrus did not decrease until 24 months of age, despite tau spreading to postsynaptic sites as early as 3 months. This spread of tau through neural circuits occurs before axon degeneration and is an early event in the disease progression [54].

miRNAs released from exosomes play a significant role in NDs, particularly AD, by modulating amyloid precursor protein (APP) and tau proteins. Dysfunctional EVs’ miRNAs may influence the progression of AD and initiate inflammation through interactions with TLR. In EV-deprived neurons, EVs’ miRNAs could regulate neuroplasticity to mitigate neurological damage. Exosomal miRNAs are stable, and their distinct profiles in various CNS diseases may serve as promising diagnostic biomarkers, even before irreversible neurological damage occurs [55]. Additionally, EVs can deliver therapeutically beneficial miRNAs across the BBB, offering potential for macromolecular drug delivery in CNS diseases. Results highlight the role of EVs’ miRNAs as diagnostic biomarkers, pathological regulators, and therapeutic agents, while also addressing challenges in their clinical applications [56,57,58]. There is further linkage to support the concept that exosomal miRNAs and mRNA can be utilized as biomarkers for AD [59,60]. Recent findings further reveal that brain-derived sEVs contain selectively packaged full-length mRNAs and that these RNA profiles are significantly altered in Alzheimer’s disease. sEVs from AD brains exhibit changes in both the abundance and composition of full-length transcripts, indicating that RNA cargo packaging is dysregulated in disease states. This suggests a potential role for full-length EV mRNAs as both indicators of pathological processes and targets for diagnostic development [60].

Additionally, some miRNAs are involved in microglia and astrocyte dysregulation, resulting in AD progression, such as miR-21, which can be transported from AD neurons and astrocytes to nearby and distant recipient cells through EVs, amplifying neuroinflammatory and neurotoxic responses [61].

Even though there is growing evidence that EV plays an important part in the tau propagation, these results should be interpreted with caution. Many mechanistic studies use artificial seeding models or overexpression systems, which might not accurately represent physiological disease states and the complex nature of the disease. Furthermore, EV-associated tau only makes up a small portion of the extracellular tau pool, and soluble, non-vesicular tau species are also known to play a significant role in intercellular transmission. Comparisons between studies can also be challenging due to variations in EV isolation and characterization techniques, and the precise vesicle subtype that causes tau transfer is frequently still unknown. When taken together, these elements suggest that tau spread is probably caused by a complex and multifaceted process, of which EV-mediated propagation is only one aspect.

## 3. Role of EVs in Diagnosis

Brain-derived EVs containing pTau181, pTau396, synaptic proteins (e.g., neurogranin, SNAP25), Aβ species, and disease-specific miRNAs have demonstrated strong predictive value for preclinical AD [62]. Distinct EV signatures also differentiate AD from other tauopathies. Emerging evidence indicates that lncRNAs, lipidomic profiles, and full-length mRNAs within exosomes may further enhance diagnostic precision. Circulating brain-derived EVs cross the blood–brain barrier (BBB), and their cargo can be measured in plasma, urine, and potentially saliva, offering minimally invasive early diagnostic alternatives to CSF sampling and neuroimaging [56,63,64,65,66]. This section focuses on the diverse molecular constituents within EVs and their diagnostic potential in tauopathies.

A wide array of EV protein and lipid biomarkers has been identified in patient biofluids. EV’s tau and Aβ proteins have been detected in plasma, CSF, and urine. Because EVs cross the BBB and reflect cellular changes in the brain, they can acquire a pathological protein signature before these proteins accumulate to detectable levels in plasma. This makes EVs a more sensitive and specific biomarker source for early AD detection [63]. Other studies reported that the molecular content of urinary EVs may assist in the diagnosis of tauopathies [66,67,68]. In parallel, several plasma EV proteins have been identified as promising biomarkers for AD [69]. Additionally, plasma EVs in AD, DLB, and FTD show reduced concentration and increased vesicle size, offering a simple biophysical signature that distinguishes patients from controls [70].

CSF biomarkers are also used for clinical diagnosis of AD and have been shown to be positively correlated with plasma neuronal-derived EVs. Thus, blood EVs’ concentrations of Aβ42, T-tau, p-S396-tau, and p-T181-tau could be used as a less invasive alternative to CSF-based screening and diagnostic tests [71,72,73]. The level of tau and TDP-43 in their plasma EVs is also beneficial for the diagnosis of other tauopathies such as frontotemporal dementia and amyotrophic lateral sclerosis [74]. In addition to phosphorylated tau species, phosphorylated insulin receptor substrate-1 (p-IRS-1) within neuronal-derived EVs (NDEV) has also been shown to predict AD years before symptom onset [75].

Among protein biomarkers, blood neuronal-derived EVs, specifically GAP43, neurogranin, SNAP25, and synaptotagmin 1, show potential as predictive biomarkers for Alzheimer’s disease, detecting it 4 to 7 years before cognitive impairment [75,76]. Moreover, plasma-derived EV Aβ1–42 was found to be superior to p-tau181 and p-tau396,404 in terms of specificity and sensitivity in AD diagnosis [77,78], with S100A8 representing another biomarker for AD in plasma EVs [79]. Neurogranin level in blood-derived EVs was identified as a cognitive biomarker for AD and mild cognitive impairment (MCI)-AD [80]. Other possible serum EV content having the potential as diagnostic biomarkers include G-protein coupled receptors (GPCRs) such as adenosine and metabotropic glutamate receptors [81]. Beyond AD, blood levels of NDEVs containing oligomeric α-synuclein and tau aggregates can accurately differentiate PD from corticobasal degeneration and PSP with high sensitivity and specificity [82,83]. Surprisingly, serum EVs derived from AD patients were found to reduce BBB integrity by affecting the VE-cadherin expression in brain microvascular endothelial cells. This effect was dependent on their RNA content [84]. Lipid-based biomarkers from plasma EVs also hold promise as diagnostic and prognostic biomarkers for AD [85]. NDEVs’ synaptic protein content can also be a measure of disease progression in AD and frontotemporal dementia, as they are decreased in this population [86].

EVs have also served as biomarkers for impaired autophagy in AD patients [87], which indicates their usefulness in clinical research and diagnosis to confirm in vivo findings, reinforcing their clinical significance as non-invasive biomarkers that reflect neuronal intracellular changes: helping in early detection, disease progression monitoring, and even evaluating therapeutic responses in AD patients [88,89,90]. Moreover, EVs’ miRNA content can serve as diagnostic biomarkers [91]; for example, miR-20, miR-132-3p, miR-122-5p, and miR-373 were downregulated in serum exosomes of AD patients compared to healthy controls [92,93,94]. However, miR-384 was upregulated in AD patients [73]. Similarly, recent studies have identified long non-coding RNAs (lncRNAs) in EVs as potential biomarkers for AD. The lncRNAs, ENST00000608936 and ENST00000433747, for instance, were differentially expressed between AD patients and controls, and had >95% sensitivity, specificity, and accuracy [95].

The ability to evaluate longitudinal predictive value is limited by the cross-sectional designs and relatively small cohorts used in all of the studies, and the small amount of longitudinal evidence. Furthermore, the variation in EV isolation techniques, enrichment strategies, and marker selection makes studies less comparable, and the interpretation of reported biomarker performance is further complicated by the difference in neuronally enriched EV fractions and total EV preparations.

Table 1 provides a summary of key studies highlighting the diagnostic potential of EV’s tau, non-tau protein biomarkers, RNA, and lipid biomarkers across different biofluid sources in Alzheimer’s disease. In a broader lens, EVs serve as significant clinical potential biomarkers in drug delivery, disease diagnosis, treatment, and other medical fields, offering unique advantages over synthetic drug delivery systems.

## 4. Role of EVs in Therapy

Because EVs can transport specific biomolecules between cells, they provide a promising platform for delivering therapeutic agents directly to affected regions of the brain. Our ability to engineer EVs for targeted drug delivery offers the potential to mitigate the spread of pathological Tau by either inhibiting its aggregation or enhancing its clearance from the brain [97,98]. EVs can also be engineered to carry therapeutic miRNAs or siRNA, proteins, or small molecules designed to modulate Tau phosphorylation, reduce neuroinflammation, or restore synaptic function, and those interventions may slow or halt the progression of tauopathies [17]. Moreover, EVs are naturally derived and display low immunogenicity, making them advantageous over synthetic delivery systems by reducing the likelihood of immune reactions. Their ability to cross the BBB further strengthens their utility for treating CNS disorders such as tauopathies [99]. With ongoing advancements in EV isolation, characterization, and engineering, these vesicles could become an essential tool for the development of personalized and noninvasive therapies, and potentially disease-modifying therapies (DMTs) for tauopathies [97]. Given the complexity of AD, single-target therapies may be insufficient, but multi-target approaches, including those targeting both tau and neuroinflammation or Aβ, are showing more promise in clinical trials. These strategies may offer breakthroughs in AD treatment, with the potential to reverse or even cure the disease.

### 4.1. EVs as Therapeutic Vehicles

EVs have gained increasing recognition as powerful therapeutic tools due to their natural ability to cross the BBB, low immunogenicity, and biocompatibility [99]. By carrying diverse biomolecules, including proteins, mRNAs, miRNAs, siRNAs, and small molecules directly to target tissues, EVs represent a promising therapeutic strategy for neurodegenerative diseases, including tauopathies [100,101]. Innovations such as exosome reporter mice and in vivo imaging techniques have greatly expanded the understanding of EVs’ biodistribution. Building on these insights, clinical trials are now evaluating exosomes as therapeutic agents [102]. In parallel, advances in cargo loading methods, including incubation, transfection, electroporation, and sonication, could expand the potential for developing new EV-based therapeutics [17,103,104,105,106].

For example, exosomes can be engineered to express specific ligands that target tau pathology, allowing for the selective delivery of neuroprotective compounds to affected neurons. This method not only improves the therapeutic index but also minimizes systemic side effects, making it a promising avenue for future research.

### 4.2. Endogenous Modulation of EVs

In addition to introducing engineered vesicles, alternative strategies focus on modulating the body’s own EV production or cargo. For example, Fasudil, a Rho-associated kinase (ROCK) inhibitor, has been shown to reduce Aβ plaque load and p-tau levels in AD models, partly through the regulation of EV miRNAs [107]. Similarly, ultrasound stimulation has been reported to enhance the release of astrocyte-derived EVs with neuroprotective properties [108]. In parallel, long-term exercise stimulates the neuronal secretion of miR-532-5p-carrying EVs, which contribute to maintaining BBB integrity [109]. Moreover, EV-mediated delivery of natural compounds such as Catalpol and Tetramethylpyrazine has demonstrated therapeutic potential by promoting neuroplasticity and regeneration through the regulation of signal transducer and activator of transcription 3 (STAT3) phosphorylation [110]. P2X7 antagonism also mitigates tau propagation by suppressing microglial EVs secretion [42].

### 4.3. Exogenous Therapeutic EVs

#### 4.3.1. Unmodified Therapeutic EVs

EVs derived from mesenchymal stem cells (MSCs), neural stem cells (NSCs), and other progenitor cells have demonstrated therapeutic effects in preclinical AD models. MSC-derived exosomes reduce glial hyperactivation and neuroinflammation while simultaneously enhancing neurotrophic support and synaptic health [96,111]. They have also been shown to stimulate neurogenesis and reduce Aβ-induced AD symptoms [112]. Similarly, NSC-derived exosomes reduce p-tau and Aβ formation and reduce neuroinflammation [113]. Exosomes from human medial ganglionic eminence progenitors further contribute to neuronal recovery by inhibiting astrocyte activation through anti-inflammatory miRNA cargo [114].

Adipose-derived mesenchymal stem cell (ADMSC) EVs have been shown to attenuate M1 microglial polarization, reduce neuroinflammation, and improve tissue repair in both AD and traumatic brain injury (TBI) [115]. Additionally, ADMSCs have been successfully employed to deliver therapeutic miRNA-22 to AD brains, mitigating pyroptosis and reducing proinflammatory signaling [116]. Complementing these findings, intranasal administration of bone marrow-derived MSC EVs has been reported to reduce Aβ plaques and glial activation while preserving BBB integrity [111,117]. Beyond MSCs, platelet-derived extracellular vesicles have also been explored for their anti-inflammatory and neuroprotective properties in Parkinson’s models [118].

Stem-cell-derived EVs represent promising alternatives to traditional cell therapies, offering reduced safety risks and greater delivery control. These vesicles aid in neuroprotection, neuroinflammation reduction, and tissue regeneration while avoiding challenges like tumorigenicity and immune rejection.

#### 4.3.2. Engineered EVs for Targeted Delivery

Therapeutic EVs can be bioengineered to enhance brain targeting and improve the delivery of disease-modifying agents [99,119]. For instance, Fe65-engineered EVs carrying Corynoxine-B (CoryB) have been shown to activate autophagy, reduce Aβ accumulation, and improve synaptic plasticity in AD models [16]. EVs can also be functionalized with targeting ligands like apolipoprotein B (ApoB) or modified via gene-editing systems. A notable example is the MAPLEX platform, which uses photoinducible intracellular delivery of gene-editing proteins to downregulate beta-secretase 1 (BACE1), thereby reducing amyloid burden [120]. EVs modification with a BBB crossing peptide and a mitochondria-targeting moiety (C3 peptide- and triphenylphosphonium (TPP)) and loading them with curcumin as the therapeutic cargo, enhanced curcumin bioavailability and rescued mitochondrial function in the neurons and downregulated pathways of neuroinflammation and improved neuronal survival [121]. Table 2 outlines both natural and engineered EV-based therapeutic strategies that have been tested in preclinical models of tauopathies and related NDs.

To clarify the distinctions between endogenous and engineered exosome applications, a comparative summary is provided in Table 3.

### 4.4. Therapeutic Potential and Remaining Hurdles

Beyond natural EVs, innovative strategies such as biomimetic EVs and liposome hybrids have been shown to improve microglial function and targeted delivery to AD lesions [122]. While these innovations mark major progress, they remain in the early stages of development. Long-term studies on safety, stability, and efficacy are still required, and the establishment of standardized protocols for EV cargo loading and consistent production remains a top priority for translating these therapies from the laboratory to clinical practice.

### 4.5. Clinical Translation of EV-Based Therapeutics

MSC-derived EVs have advanced past preclinical stages and are now being tested in early-phase clinical trials to treat ND and tauopathies. Using intranasal MSC-derived EV therapies’ low immunogenicity, neuroprotective cargo, and blood–brain barrier-crossing capabilities, a number of ongoing and completed trials have shown their safety and encouraging early efficacy. For example, intranasal delivery of allogeneic adipose MSC-derived EVs was tested in a Phase I/II trial in AD patients (NCT04388982), which reported no side effects and mild cognitive benefits in addition to hippocampal structural preservation [102]. Similarly, AD, PD, FTD, and ALS are being studied using human umbilical cord-derived MSC EVs (NCT06607900, NCT05152394, NCT06598202) [123,124,125]. The goal of these therapeutics is to directly administer neurotrophic and anti-inflammatory substances to the central nervous system via nasal drops.

Moreover, early-phase studies have demonstrated functional improvements and slowed progression in ALS Lewy Body dementia, Kennedy disease, and Congenital Myasthenic Syndrome (CMS) patients treated with AlloEx Exosome^®^ therapy: an MSC secretome enriched for EVs and bioactive molecules (NCT07105371) [126]. Nevertheless, targeted delivery, large-scale production, and regulatory standardization remain current challenges. These advancements show the growing trend toward EV-based precision therapies.

## 5. Challenges and Limitations in Clinical Applications

Despite rapid progress, significant barriers limit the clinical translation of EV-based diagnostics and therapeutics. Isolation methods vary widely in yield and purity, complicating standardization across studies. Cargo loading techniques remain inconsistent and may affect vesicle stability or biological activity [127,128,129]. EVs are rapidly cleared by the mononuclear phagocyte system, necessitating surface modifications such as PEGylation or CD47 expression to extend circulation time. Regulatory approval requires rigorous characterization, potency assays, and reproducible manufacturing pipelines that comply with evolving ISEV and FDA guidance. Balancing suppression of pathological EV-mediated tau spread with preservation of physiological EV signaling remains an additional therapeutic challenge.

### 5.1. Isolation, Standardization, and Yield

A major limitation lies in the absence of standardized and scalable methods for EV isolation [130]. Current methods, such as ultracentrifugation, size-exclusion chromatography, and precipitation, often produce variable yields, purity levels, and cargo compositions [97,131]. Human error and methodological inconsistencies further hinder the validation of findings and reproducibility across studies [130]. While microfluidic isolation platforms offer improved control and sensitivity, their limited sample capacity presents a trade-off. Ongoing efforts are focused on developing integrated devices capable of achieving high yield, purity, time-saving, and cost-efficiency simultaneously, which will be essential for advancing EV-based applications towards clinical use [132,133]. Notably, acoustic trapping emerged as a promising approach to isolate EVs from low-volume samples, with minimal preprocessing. It was shown to be successful in isolating EVs from small CSF samples for tau biomarker analysis in AD [134]. Moreover, an immunomagnetic exosomal PCR platform was introduced by Hu et al. (2024) that detects blood exosomal Aβ1-42, p-tau181, and p-tau396/404 with femtogram sensitivity [77].

### 5.2. Cargo Loading and Engineering Challenges

Another major hurdle involves achieving efficient and consistent cargo loading. Current methods, including incubation, electroporation, sonication, and freeze–thaw cycles, vary widely in efficiency and often risk altering EV membrane integrity or cargo stability [135]. Choosing the appropriate strategy often depends on the nature of the cargo, the target tissue, and the desired pharmacokinetic profile [127]. More advanced methods, such as surface ligand functionalization or genetic engineering of donor cells, hold promise but require additional safety validation to address concerns about off-target effects and transfection-related toxicity [136]. We demonstrated the fast EV loading and delivery of protein [119,137], 3′-UTR fragment [138] and short hairpin RNA [139], the last of which received FDA approval as an orphan drug for brain tumors (https://www.accessdata.fda.gov/scripts/opdlisting/oopd/detailedIndex.cfm?cfgridkey=972423 (accessed on 23 September 2025)).

### 5.3. Biodistribution, Pharmacokinetics, and Clearance

Exosomes also face rapid clearance by the mononuclear phagocyte system (MPS), which limits their half-life and therapeutic window. To overcome this limitation, strategies such as PEGylation or CD47 surface expression have been developed to evade immune recognition and prolong circulation [127,140]. Additionally, tumor- or CNS-targeting ligands, including peptides, aptamers, and antibodies, have shown promise in enhancing delivery specificity [141]. Incorporating stimuli-responsive elements, such as pH sensitivity, ultrasound, or magnetic guidance, offers further opportunities for controlled delivery and site-specific release [108,142,143].

### 5.4. Regulatory and Ethical Barriers

The complexity of EV production also raises ethical and regulatory concerns. Variability in production protocols affects batch consistency, complicating quality control and FDA approval processes [100,127,144,145]. Moreover, ethical issues regarding source material, particularly stem-cell-derived EVs, and uncertainties surrounding long-term biosafety must be thoroughly evaluated before clinical implementation.

### 5.5. Exosome Function and Tau Propagation Specificity

Therapies targeting EV-mediated tau propagation must balance effectiveness with the preservation of physiological exosome functions. Evidence indicates that tau protein release via EVs is threshold- and environment-dependent. Although preferential release at synaptic terminals represents only a small fraction of extracellular tau, it plays a crucial role in driving tau spread [53].

EVs, particularly exosomes, can leak from multivesicular bodies and bypass degradation pathways, complicating efforts to prevent pathological spread without impairing normal intercellular communication [36,146]. This is supported by the fact that tau-loaded EVs are enriched in lysosomal substrates and are secreted due to lysosomal dysfunction [23]. Microglial EVs are particularly essential for immune surveillance and myelin regeneration in the CNS, making them difficult to inhibit without disrupting key protective processes [147,148].

Future research should prioritize refining EVs engineering strategies to improve targeting and minimize unintended effects. Approaches may include genetic editing tools or ligand decoration to restrict exosome function to pathological zones. Complementary approaches such as small molecule inhibitors or immunotherapies may further support tau clearance while preserving immune homeostasis. With improved strategic innovation and cross-disciplinary collaboration, exosomes hold promise as precise, safe, and scalable platforms for treating tauopathies and related NDs.

## 6. Conclusions

Substantial advancements have been made in research relating to EVs and tauopathy. EVs have been increasingly recognized as central mediators of tau propagation, particularly through microglial pathways, while also offering an opportunity for biomarker discovery and early disease detection.

EVs also constitute a promising therapeutic approach for tauopathies due to their low immunogenicity, ability to cross the BBB, and ability to deliver a variety of cargoes directly to affected neurons. Both endogenous modulation and exogenous approaches using stem-cell-derived or engineered EVs have shown promise. Targeting accuracy and therapeutic efficacy are further enhanced by engineered EVs, such as ligand-targeted vesicles and gene-editing platforms.

However, the challenges in effectively utilizing EVs for targeted delivery and therapeutic purposes remain substantial, including issues with isolation, stability, and efficient targeting. Emerging strategies to enhance EV function and neuroprotective capabilities hold great promise, yet a deeper understanding of their mechanisms and interactions with tau aggregates is essential. Future research is critical to refine these methods, overcome existing barriers, and fully harness the therapeutic potential of EVs. Only with continued investigation can we truly realize their capacity to alter the course of tauopathies and bring novel treatments to reality.

## Figures and Tables

**Table 1:** A summary of representative studies evaluating EV’s tau, miRNAs, mRNAs, and proteins as diagnostic biomarkers across biofluids, including CSF, plasma, and urine.

	Study	Design/Methodology	Disease Model	Source of EVs	Key Findings
Tau-Related EV Biomarkers	Saman et al. 2012 [12]	Experimental tauopathy models + human CSF analysis	AD	CSF, neurons	EV’s tau phosphorylated at Thr-181 was elevated in AD CSF and indicates early diagnostic potential.
	Cano et al. 2023 [63]	Cross-sectional study	Early-onset MCI	CSF, Plasma	EV p-T181 is elevated in early-onset MCI, and correlated with hippocampal atrophy and cognitive decline
	Sun et al. 2019 [67]	Case–control pilot study	AD	Urine	Aβ1-42 and P-S396-tau are enriched in AD-derived urinary EVs
	Li et al. 2024 [68]	Case–control study	PSP	Urine	Elevated p-T181-tau neural cell markers in PSP-derived urinary EVs
	Jia et al. 2019 [71]	Multicenter, two-stage clinical study	AD	CSF, Plasma	Elevated Aβ42, T-tau, and P-T181-tau levels in AD-derived EVs
	Fiandaca et al. 2015 [72]	Case–control study + longitudinal	AD, FTD	Plasma, Serum	AD: elevated total tau, P-T181-tau, P-S396-tau and Aβ1-42. FTD: elevated p-T181-tau and Aβ1-42 AD-derived EVs show changes up to 10 years before diagnosis
	Chatterjee et al. 2024 [74]	Cross-sectional biomarker discovery and validation study	FTD, FTD spectrum disorders, ALS	Plasma	high EV TDP-43 in ALS and TDP-43+ bvFTD; low EV 3R/4R tau ratio in PSP, high in tau+ bvFTD
Non-Tau Protein EV Biomarkers	Jia et al. 2021 [76]	Two-stage cross-sectional and longitudinal validation studies	AD	Plasma	Lower levels of synaptic proteins (GAP43, neurogranin, SNAP25, synaptotagmin-1) in AD; predicted AD 5–7 years before symptoms
	Goetzl et al., 2016 [86]	Cross-sectional and longitudinal study	AD, FTD	Plasma	EV- levels of synaptophysin, synaptopodin, synaptotagmin-2, and neurogranin were reduced in AD and FTD; changes detected years before dementia onset.
	Hu et al. 2024 [77]	Clinical validation study	AD	Blood	Blood EV Aβ1–42 has higher specificity and sensitivity for AD diagnosis compared to p-tau181 and p-tau396,404
	Cai et al. 2022 [69]	Case–control pilot study	AD	CSF, Plasma	↑A0A0G2JRQ6), C1QC, CO9, GP1BB, RSU1; ↓ disintegrin and ADA10
	Jiang et al., 2021 [83]	Multi-cohort study	PD, MSA, PSP, CBS	Serum	EV- α-Synuclein and clusterin provide high diagnostic accuracy of PD from other movement disorders
	Meloni et al. 2023 [82]	Case–control study	PD, CBD, PSP	Serum	Oligomeric α-synuclein ↑ in PD; tau aggregates ↑ in CBD, PSP and these were correlated with disease severity
	Zhang et al. 2024 [79]	Case–control and in vitro validation	AD	Plasma	S100A8 is a ↓EV protein in AD
	Kapogiannis et al., 2019 [75]	Case–control study with longitudinal validation	AD	Plasma	↑p-tau181, p-tau231, and phosphorylated IRS-1 predicted AD up to 4 years before onset
	Eitan et al., 2023 [62]	Methodology validation + case–control study	AD	Plasma	↑ p181-Tau, Aβ42, neurogranin; ↓proBDNF, GluR2, PSD95, GAP43, and Syntaxin-1 in AD; classifier model correctly identified 94.7% of AD cases
	Longobardi et al., 2021 [70]	Case–control study	AD, DLB, FTD	CSF	AD and DLB had ↑ EV concentration and ↓ EV size; EV size best discriminated patients from controls; correlated with p-tau181/Aβ42 ratio
	Eren et al., 2022 [88]	Cross-sectional study	AD	Plasma	Higher NDEV Aβ42 and synaptic proteins linked to better cognition
	Cai et al., 2024 [66]	EV surface protein profiling via proximity barcoding assay; machine learning-based classification	AD	Urine, plasma, saliva, tears, and serum	Urinary EVs showed highest diagnostic power; specific EV subpopulation (PLAU^+^/ITGAX^+^/ANXA1^+^) predicted AD with 88% accuracy
	Yuyama et al., 2024 [89]	Quantitative proteomics	AD	CSF, Plasma	Cathepsin B in EVs was significantly altered across ATN stages; EV CatB levels inversely correlated with CSF Aβ42
	Muraoka et al., 2020 [90]	Pilot case–control proteomic study	AD, MCI	CSF	Identified 3 proteins (HSPA1A, NPEPPS, PTGFRN) linked to AD progression; PTGFRN correlated with amyloid plaques and tau tangles in CSF EVs.
	Boyer et al., 2024 [78]	Case–control study	AD	Plasma	Soluble Aβ42/40 outperformed EV-associated biomarkers
RNA and Lipid EV Biomarkers	Li et al. 2022 [73]	Multicenter, two-stage study	AD, aMCI,	Plasma	Elevated Aβ42, Aβ42/40, tau, P-T181-tau, and miR-384 in aMCI and AD; strong correlation with CSF levels; dual-labeled EVs showed diagnostic potential for early-stage AD.
	McKeever et al., 2018 [65]	Case–control study	Young onset AD	CSF	Altreed EV-miRNA profiles in YOAD; miR-125b-5p, miR-451a, and miR-605-5p linked to tau pathology and neuroinflammation identified
	Mosquera-Heredia et al., 2024 [95]	Case–control study	AD	Serum	ENST00000608936, ENST00000433747 lncRNA have high diagnostic accuracy
	Krokidis et al. 2024 [85]	Case–control study	AD	Plasma	Distinct lipid species (Cer, PC, LPE) were differentially expressed in AD
	Liu et al., 2020 [96]	Meta-analysis	AD, MCI	CSF, Plasma	Pasma EV-Ng is ↓in AD & MCI-AD
	Visconte et al., 2023 [59]	Case–control with validation cohort	Prodromal AD	Plasma	Plasma EVs showed widespread dysregulation of miRNAs; miR-16-5p, miR-25-3p, miR-92a-3p, and miR-451a were significantly ↑in prodromal AD
	Reho et al., 2025 [91]	Case–control study	AD, preclinical AD	Serum	Identified 14 miRNAs associated with AD; preclinical AD showed more pronounced miRNA changes than clinical AD; miRNA targets included SNCA, CYCS, MAPT and other neurodegeneration-related genes
	Sbriscia et al., 2025 [93]	Case–control study	Mild & moderate AD, MCI	Plasma	miR-132-3p was ↓in AD; ↑in MCI; correlated with plasma levels; potential early biomarker
	Subasinghe et al., 2025 [94]	Case–control study	Cognitive Impairment, AD	Plasma	miR-122-5p was ↓ in cognitively impaired individuals

↑ indicates increased/upregulated levels; ↓ indicates decreased/downregulated levels. Abbreviations: Aβ, amyloid-β; Aβ1–42, amyloid-β 1–42; Aβ42/40, amyloid-β 42/40 ratio; AD, Alzheimer’s disease; ALS, amyotrophic lateral sclerosis; aMCI, amnestic mild cognitive impairment; α-syn, α-synuclein; bvFTD, behavioral variant frontotemporal dementia; CBD, corticobasal degeneration; CBS, corticobasal syndrome; Cer, ceramide; CSF, cerebrospinal fluid; EVs, extracellular vesicles; FTD, frontotemporal dementia; GAP43, growth-associated protein 43; iPSC, induced pluripotent stem cell; LPE, lysophosphatidylethanolamine; lncRNA, long non-coding RNA; MCI, mild cognitive impairment; miRNA, microRNA; MSA, multiple system atrophy; Ng, neurogranin; PC, phosphatidylcholine; PD, Parkinson’s disease; p-tau, phosphorylated tau; p-S396-tau, tau phosphorylated at serine-396; p-T181-tau, tau phosphorylated at threonine-181; PSP, progressive supranuclear palsy; SNAP25, synaptosomal-associated protein 25; TDP-43, TAR DNA-binding protein 43; T-tau, total tau; YOAD, young-onset Alzheimer’s disease; 3R/4R tau, three-repeat/four-repeat tau isoforms.

**Table 2:** An overview of therapeutic exosome strategies used in preclinical models of Alzheimer’s disease and related tauopathies.

Study	Therapy Type	Disease Model	Source of EVs	Engineered	Key Findings
Ruan et al., 2020 [42]	Small molecule-induced modulation	P301S Tau Mouse	Microglia(via P2RX7)	Indirect	GSK1482160 reduced tau EVs release and improved overall cognition
Chen et al., 2023 [51]	Photo modulated microglial exosomes	AD Mouse	M2 microglia + 1070 nm light	Induced	The miR-7670-3p-enriched EVs reduced overall ER stress and inflammation
Zhao et al., 2023 [97]	Endogenous (natural exosomes)	AD	APP cleavage-related exosomes	No	Exosomes carry APP/Tau cleavage products, meaning they could modulate the pathology
Liu et al., 2022 [96]	MSC-derived exosomes	AD Mouse	Bone marrow MSCs	No	There was improved brain-derived neurotrophic factor (BDNF) signaling and reversal of cognitive decline
Han et al., 2024 [120]	MAPLEX Platform	AD	Custom CRISPR-Cas cargo	Yes	Epigenome editing was shown to reduce BACE1 expression and amyloid burden
Lyaswamy et al., 2023 [16]	Corynoxine-B	AD mice	hippocampus neurons	Yes	Targeted delivery of Corynoxine B to affected neurons
Geng et al., 2023 [15]	FTO-targeted siRNA	PD mice	MSCs	No	Reduced dopaminergic neurodegeneration
Ding et al., 2025 [121]	Curcumin	tau^p301s^ mice	Plasma	Yes	Targeted delivery of curcumin, reduced p-tau, oxidative stress, and tangles; improved mitochondrial function

Abbreviations: AD, Alzheimer’s disease; APP, amyloid precursor protein; BACE1, beta-secretase 1; BDNF, brain-derived neurotrophic factor; CRISPR, clustered regularly interspaced short palindromic repeats; ER, endoplasmic reticulum; FTO, fat mass and Obesity-associated gene; MSC, mesenchymal stromal cell; P2RX7, P2X purinoceptor 7.

**Table 3:** A comparison of the advantages, challenges, and clinical considerations of endogenous (natural) vs. engineered EVs.

Category	Definition	Advantages	Challenges	Examples
Endogenous	Natural vesicles	Excellent biocompatibility and low immunogenicity Intrinsic cargo reflecting parent cell status Natural ability to cross biological barriers (e.g., BBB) Suitable for autologous use	Heterogeneous populations with variable cargo Limited control over targeting and loading Low yield and scalability issues	MSC-, NSC-, microglia-derived EVs
Engineered	Modified to carry specific cargo or ligands	Targeted delivery via engineered ligands Customizable cargo (e.g., RNA, proteins, CRISPR) Potential to overcome natural delivery limitations Enhanced CNS penetration and retention	Complex production and regulatory hurdles Risk of immunogenicity from engineered components Limited in vivo tracking and dose control High cost of manufacturing	CRISPR-loaded exosomes, ApoB-targeted vesicles

Abbreviations: ApoB, Apolipoprotein B; MSC, mesenchymal stromal cell; NSC, neural stem cells.

## Data Availability

No new data were created or analyzed in this study. Data sharing is not applicable.

## Abbreviations

Aβ, amyloid-β; α-syn, α-synuclein; AAV, adeno-associated virus; AD, Alzheimer’s Disease; ADEV, astrocyte-derived exosome; ALS, amyotrophic lateral sclerosis; Apolipoprotein B, ApoB; APP, amyloid precursor protein; ADMSC, adipose derived mesenchymal stem cells, BACE1, beta-secretase 1; BBB, blood–brain barrier; BDNF, brain-derived neurotrophic factor; BIN1, bridging integrator 1; CBD, corticobasal degeneration; CD47, Cluster of Differentiation 47; CNS, central nervous system; CSF, cerebrospinal fluid; DAM, disease-associated microglia; DMTs, disease-modifying therapies; ER, endoplasmic reticulum; EVs, extracellular vesicles; GPCR, G-protein coupled receptors; IL-1β, interleukin-1β; INSR, insulin receptor; ISEV, international society of extracellular vesicles; MISEV, minimal information for studies of extracellular vesicles; MPS, mononuclear phagocyte system; MSC, mesenchymal stromal cell; MyD88, myeloid differentiation primary response; n2a cells, neuro-2a cells; ND, neurodegenerative disorder; NDEV, neural-derived extracellular vesicles; NSC, neural stem cells; P2RX7, P2X purinoceptor 7; PCR, polymerase chain reaction; PD, Parkinson’s Disease; PART, primary age-related tauopathy; PEG, polyethylene glycol; PSP, progressive supranuclear palsy; NCAM, neural cell adhesion molecule; sEV, small extracellular vesicles; STAT3, signal transducer and activator of transcription 3; Thr-181, threonine 181; TLR, toll-like receptor; TREM2, Triggering receptor expressed on myeloid cells 2; TBI; traumatic brain injury.
